# A Combined Approach of Field Data and Earth Observation for Coastal Risk Assessment

**DOI:** 10.3390/s19061399

**Published:** 2019-03-21

**Authors:** Maria Francesca Bruno, Matteo Gianluca Molfetta, Luigi Pratola, Michele Mossa, Raffaele Nutricato, Alberto Morea, Davide Oscar Nitti, Maria Teresa Chiaradia

**Affiliations:** 1 Department of Civil, Environmental, Building Engineering and Chemistry, Technical University of Bari—Via E. Orabona, 4, 70125 Bari, Italy; matteogianluca.molfetta@poliba.it (M.G.M.); luigi.pratola@poliba.it (L.P.); michele.mossa@poliba.it (M.M.); 2 Geophysical Applications Processing (GAP) s.r.l., 70126 Bari, Italy; raffaele.nutricato@gapsrl.eu (R.N.); alberto.morea@uniba.it (A.M.); davide.nitti@gapsrl.eu (D.O.N.); 3 Department of Physics “M. Merlin”, Technical University of Bari, 70126 Bari, Italy; mariateresa.chiaradia@ba.infn.it

**Keywords:** integrated coastal zone management, coastal risk, shoreline erosion, Earth Observation, COSMO-SkyMed, ground truths

## Abstract

The traditional approach for coastal monitoring consists in ground investigations that are burdensome both in terms of logistics and costs, on a national or even regional scale. Earth Observation (EO) techniques can represent a cost-effective alternative for a wide scale coastal monitoring. Thanks to the all-weather day/night radar imaging capability and to the nationwide acquisition plan named MapItaly, devised by the Italian Space Agency and active since 2010, COSMO-SkyMed (CSK) constellation is able to provide X-band images covering the Italian territory. However, any remote sensing approach must be accurately calibrated and corrected taking into account the marine conditions. Therefore, in situ data are essential for proper EO data selection, geocoding, tidal corrections and validation of EO products. A combined semi-automatic technique for coastal risk assessment and monitoring, named COSMO-Beach, is presented here, integrating ground truths with EO data, as well as its application on two different test sites in Apulia Region (South Italy). The research has shown that CSK data for coastal monitoring ensure a shoreline detection accuracy lower than image pixel resolution, and also providing several advantages: low-cost data, a short revisit period, operational continuity and a low computational time.

## 1. Introduction

Sea and the coastal areas constitute a driving force for the economic activity based on maritime resources, the so-called “blue economy” [1]. Actually, maritime and coastal tourism are the leading sectors of the European maritime economy. The priority objective of the coastal policy should hence be to enhance ecological and landscape resources in order to further increase their attractiveness and economic and social growth. Any enhancing measure strongly requires proper governance of coastal areas through integrated management plans and actions targeted to mitigate and counteract coastal risks [2]. Therefore, a detailed outline of the dynamics of coastal erosion phenomena over different time-scales is necessary for an effective coastal risk assessment and for land-use planning purposes [2,3].

Growing urbanization, human activities and climate change are exacerbating the impact of shoreline erosion phenomena [4]. Hence, shoreline position and its variability over time are an essential element for coastal management [5,6], coastal protection structure evaluation [7], validation and calibration of numerical models [8,9,10] and climate change studies [11,12]. The coastline can ideally be defined as the land-sea interface [13], and its position is constantly changing over time, mainly due to the dynamic nature of the sea level that is influenced by astronomical tides, waves, storm surge, set-up, run-up and sediment transport (cross-shore and long-shore currents) [14,15]. Therefore, the shoreline position must be defined in the most appropriate temporal scale; in the case of a long-term study, a yearly coastline can be adequate [12], whereas in the case of run-up analysis, it may be necessary to sample positions with much greater frequency [9,16].

In situ measurement techniques have been successfully adopted for shoreline observation obtaining accurate results, such as the ones that involve Global Navigation Satellite System (GNSS) campaigns [5,17,18], but a continuous GPS monitoring turns out to be impractical in terms of costs and logistics over a wide scale.

Innovative monitoring approaches, based on remote sensing techniques, could be an economically sustainable alternative providing information on shoreline evolution trend and promptly indicating also the occurrence of risk situations [19]. Different remote sensing measurement techniques can be successfully adopted for shoreline observation: optical sensors on both aerial and space platforms [19], LiDAR campaigns [20,21], Unmanned Aerial Vehicle (UAV) [22,23,24,25], videomonitoring systems [26,27,28,29]. The exploitation of very-high resolution aerial optical images and LIDAR surveys allows very accurate positioning of the shoreline over a wide scale, but their high acquisition costs strongly limit the frequency of monitoring, whereas small UAVs and videomonitoring systems offer a low cost solution but they are able to provide information only about limited areas.

Over the years, Synthetic Aperture Radar (SAR) has proved to have a positive cost-benefit ratio for territorial analysis, from small/medium to wide scale and the all-weather day/night imaging capability has made this approach very popular, especially for mapping remote areas or under extreme climatic conditions [19]. The use of satellite SAR sensors has been limited in the past by the low spatial resolution of the images [30,31]. Since 2007, the new constellations of satellites equipped with high resolution X-band SAR sensors, such as the COSMO-SkyMed (CSK) and TerraSAR-X (TSX) missions, potentially allowed to identify and monitor shoreline evolution with meter or sub-meter level accuracy (according to the selected acquisition mode), thus fostering the exploitation of SAR data for coastline extraction. Moreover, the nationwide acquisition plan named MapItaly has been devised by the Italian Space Agency (ASI), to provide CSK high-resolution satellite SAR images covering the whole Italian territory with a 16-day revisit period [32].

The existing approaches for shoreline extraction from SAR images are classified into two types: radiometric approach, certainly the most used, and interferometric approach. The first approach attempts to locate the discontinuities present in significant intensity values of the images [33,34,35,36] whereas the second exploits the loss of coherence typical of water-covered areas [37,38]. To overcome the issues related to the presence of noise in SAR images, numerous other approaches, based on complex mathematical models such as Active Contour Model [39,40], Level Set Functions [41,42,43,44], Snake models [45] have been tested with good results over large areas.

In the last few years, the very fine spatial and radiometric resolution of COSMO-SkyMed SAR images has encouraged the development of several procedures aimed at shoreline extraction. CSK amplitude SAR image processing [46,47] has provided very satisfactory results in coastline mapping achieving an accuracy equivalent to the spatial resolution, proved by comparing results with in situ shoreline measures acquired during satellite observation. A Bayesian estimation theory able to detect sea boundaries at full resolution and low error rate in a totally unsupervised way has also been proposed [48].

An interferometric technique for the coastline extraction from CSK interferometric pairs, using the coherence loss of pixels belonging to the sea surface, has been developed [38]. Dual polarimetric CSK data for coastline extraction have also been experimented [49,50,51], exploiting the correlation between co- and cross-polarized amplitude channels. Furthermore, an innovative coastline extraction procedure from Full-Polarized SAR imagery has been developed to integrate an Autoassociative Neural Network (AANN) and a Pulse-Coupled Neural Network (PCNN) [52].

Field data are always essential to improve the robustness, accuracy and reliability of any approach for coastal risk assessment based on Earth Observation (EO) data. In particular, remotely sensed data must be accurately geocoded, validated and corrected on the basis of the actual marine weather conditions.

This paper presents a semi-automatic integrated approach for shoreline detection and monitoring, as well as its application on two different test sites in Apulia Region (South Italy). An innovative model combining backscatter intensity data with the interferometric analysis has been tested on CSK Stripmap HIMAGE mode data providing an accurate shoreline detection and a coastal type classification. Thanks to the integration of satellite SAR images and ground truths (that are used for proper EO data selection, tidal corrections and validation of EO products), the developed processing chain provides a complete SAR image analysis system for shoreline monitoring, allowing to overcome some issues in the processing of SAR data and ensures the best possible results in coastal monitoring, as remarked in the following sections. The paper is structured as follows: Section 2 provides details on the developed integrated EO system and its outlines; Section 3 illustrates the features of the case study and available data and in Section 4 the obtained results are presented. Section 5 contains the discussion on the proposed scheme and in Section 6, conclusions are provided.

This paper is an extended version of “Remote Sensed and In Situ Data: an integrated approach for Coastal Risk Assessment” published in “2018 IEEE International Workshop on Metrology for the Sea; Learning to Measure Sea Health Parameters (MetroSea) Conference” Proceedings [53].

## 2. Methodology

The principle behind all applications of SAR intensity data to shoreline extraction is based on the active nature of the sensor: smooth surfaces reflect incident radiation mainly in the specular direction, while rougher surfaces have a more diffuse scattering pattern. Therefore, backscattering level is negligible on calm water bodies with respect to land areas. This suggests immediately the use of a threshold approach to discriminate land from water within a SAR image. However, precise shoreline extraction from SAR images is a non trivial task since several factors influence the actual backscattering level, including surface roughness and soil moisture (for land areas), and the presence of capillary waves (for water bodies). Moreover, SAR images are affected by speckle noise that causes statistical fluctuations in the backscattering levels, hindering stable detection of threshold values.

Many methods have been developed to deal with the shoreline extraction from SAR data. They generally consist of two steps: (i) despeckling; (ii) segmentation of the SAR image through the identification of the land-sea interface.

Concerning the first step, it can be performed in different ways, i.e., through spatial or temporal filtering operators, or by exploiting polarization diversity. Since the aim of our research has been to design an operational EO system for the continuous monitoring of coastal areas, full polarized data have not been considered because the MapItaly program routinely collects only Stripmap HIMAGE single-polarization acquisitions. The adaptive despeckling algorithm proposed by [54] has been adopted in this study, since it has been demonstrated to provide a satisfactory trade-off between noise reduction and edge sharpness.

The traditional techniques for land-sea image partitioning exploit edge-detection operators [55,56], generally gradient-based, that, although effective, are not very robust, since they are not based on explicit mathematical models of speckle noise and edge extraction. The “classic” edge-detection algorithms are very sensitive to noise and, consequently, their effectiveness depends on the filters used to reduce noise to acceptable levels. However, too powerful filters can blur the edges; so, in order to obtain satisfactory results it is necessary to find a fair compromise between the noise reduction and edge sharpness. In addition, the choice of threshold values for use in edge-detection algorithms represents a crucial point, due to the risk of including some spurious elements, not belonging to the land-sea interface.

In this paper different segmentation techniques have been experimented for land-sea interface extraction from SAR images and an innovative algorithm, based on active contour analysis, has been tested [57]. A performance evaluation of the innovative algorithm has been carried out by comparing the extracted shorelines to GPS shorelines measured simultaneously with the SAR acquisition.

**Figure 1:**
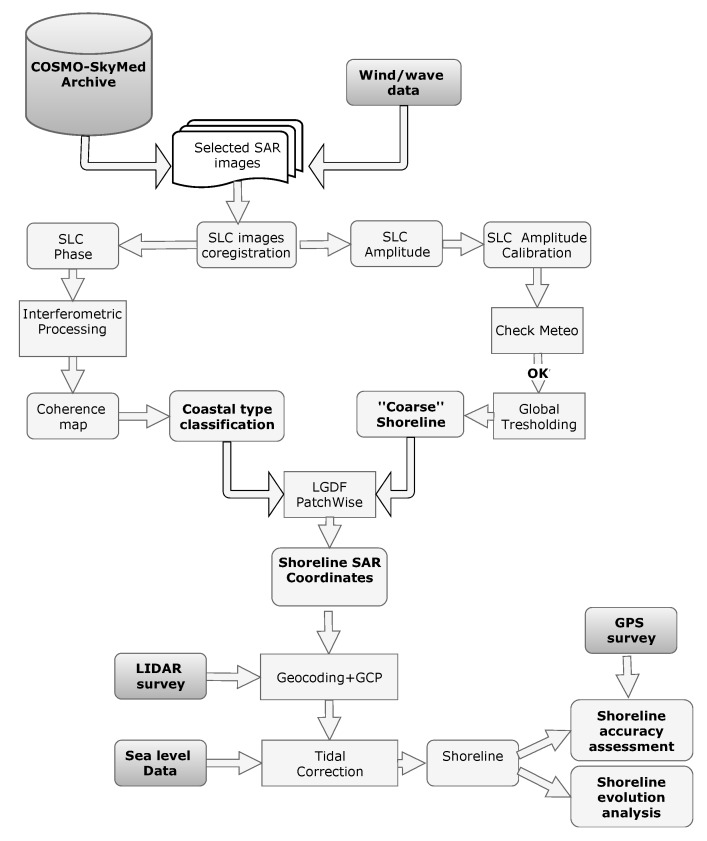
Diagram flow of the COSMO-Beach coastal monitoring system.

Moreover, as part of the experimental investigation, a data processing chain, known as COSMO-Beach [58,59], has been implemented to perform shoreline monitoring using both SAR amplitude and interferometric coherence information [60]. The developed system exploits remote sensed images from CSK constellation combined with detailed topographic and meteorological data collected in situ (Figure 1). The implemented procedure consists in (i) the selection of satellite images to be included in the processing, based on automatic checks of the marine weather conditions over the target area, (ii) the morphological classification of the coastline, (iii) the segmentation of SAR data for land-sea interface identification and (iv) the final tidal correction. Following these steps, a diachronic analysis of the extracted shorelines can be achieved. Hereafter, the main processing steps are outlined.

### 2.1. Marine Weather Conditions

Wind and wave conditions during the SAR acquisition greatly influence the backscattering on the sea surface. In the presence of waves, the values of the backscattering coefficient ($\sigma_{0}$) are much higher than under calm conditions. In particular, X-band SAR data seems to be more sensitive to the waves compared to L-band SAR [61].

Strong wave motion can lead to erroneous definition of the coastline, which would be landward by several pixels compared to the real position [39]. Therefore, sea conditions are an essential element to avoid selecting “noisy” images, whose processing could produce unreliable results and large errors in shoreline extraction.

The exploitation of wind and wave data allows a preliminary selection of the SAR images archived in the catalogue, and to discard *a priori* all the images acquired during storms or immediately following them. Post storm images, indeed, could be useful for the quantification of damage, but they could lead to a wrong assessment of coastal dynamics. Since wave buoys are often too sparsely distributed along the coastal zone and very few coastal areas are provided with local wind and wave measures, an analysis has been carried out aimed at extracting information on sea conditions directly from SAR data amplitudes, to be exploited in the case of unavailability of in situ measurements. Wind and wave fields can be directly inferred from SAR amplitude through a number of complex algorithms [62,63,64]. Actually, we are not interested in the estimation of the wave field, but rather in selecting only the SAR images acquired under calm weather conditions. Therefore, a simplified procedure has been proposed and implemented in the COSMO-Beach processing chain that consists in a first-order statistical analysis, based on the computation of mean and standard deviation of backscattered amplitude data in open sea areas located offshore the target regions.

### 2.2. Coastal Type Classification

A further key element of the MapItaly program is to provide SAR images taken from slightly different observation directions, thus allowing SAR interferometric (InSAR) analyses, based on the coherent nature of SAR imaging.

InSAR analysis has been applied in this study, since the estimation of the interferometric coherence through ergodic estimators can provide an additional information layer to be used for remote classification of the coastal morphology. This allows in turn: (i) the automatic identification of rocky and sandy coastal areas, (ii) the adaptive and optimal configuration of the segmentation algorithm, thus ensuring satisfactory performances regardless of the coastal type of the investigated areas.

A further preliminary step consists in the coastal type classification, which is recommended since it can enhance the land-sea boundary extraction. The next processing step, consisting in the segmentation of the SAR images, must be indeed properly and differently configured for sandy and rocky segments of the coast.

The COSMO-Beach processing chain automatically detects the shoreline type, by taking into account both amplitude and interferometric coherence information: high coherence and signal amplitude values allow to classify image pixels as “rock” or “coastal structure”, while the pixels with lower values are recognized as “sand”. The selected SAR dataset is first co-registered with sub-pixel accuracy [65] and an average coherence map is hence computed from multiple interferometric pairs. Optimal co-registration results are obtained by properly selecting a reference image named Master. More specifically, the perpendicular baselines and dates of the available acquisitions are analyzed in order to identify the acquisition closer to the “mass center” of the point cloud representing the various images in the space of temporal and perpendicular baselines. This selection is performed automatically although it can be changed by the user in those cases in which the automatic result is not adequate due to the presence of strong artifacts (e.g., atmospheric or focusing artifacts).

Furthermore, SAR amplitude images are radiometrically calibrated and despeckled. In order to mask out ambiguities and artifacts, apodization filters are also recommended [66].

Available ortophotos are, hence, used for the tuning of the amplitude and coherence thresholds to be configured in the coastal classification process.

### 2.3. Land-Sea Interface Extraction

A radiometric approach has been used for the extraction of the shoreline: it exploits the amplitude of SAR images by assuming that, in the absence of wave and/or wind, surface water exhibits very low backscattering values with respect to onshore areas.

Three different segmentation approaches have been implemented and tested on real data: (i) edge-detection methods, (ii) region-growing algorithms, (iii) active contour analyses. The first two approaches have been widely experimented for shoreline extraction from SAR images, with satisfactory results. The last one, known as Local Gaussian Distribution Fitting (LGDF) [57], is an innovative approach, originally developed for bio-medical applications, and never experimented before for the segmentation of SAR images. The LGDF approach has been tested because SAR images have a similar behavior as medical diagnostic images that are also affected by many noise sources and classical edge- or region-based segmentation approaches often fail.

In order to assess the potentials of shoreline extraction algorithms based on high resolution SAR data acquired by the CSK constellation, tests have been performed on both sandy and rocky (or urbanized) shores. The LGDF algorithm has been proved to work well in all the examined situations and it has been implemented in a two-step shoreline extraction process. The co-registered dataset is initially segmented using a global thresholding algorithm [55], in order to extract a preliminary “coarse” shoreline. The land-sea interface detection is hence refined by exploiting the “first-guess” land-sea boundary as well as the coastal type information, that are both provided as input to the LGDF segmentation code.

After the LGDF segmentation step, for noisy images, the end result might not be what is expected and a refinement step must be performed. In such cases, COSMO-Beach enables the operator to re-analyze SAR images and manually change input parameters of the segmentation algorithm. This change can be applied globally (i.e., to the whole image) or locally, i.e., only on those stretches that exhibit critical results, thus allowing a faster and more accurate refinement. In any case, a visual inspection of segmentation results by a skilled operator is recommended, although time-consuming, since land-sea interface from SAR images is not clear as shoreline from optical images. The visual inspection and the refinement step require operator interaction, whereas all the other processes are fully automated.

### 2.4. Precise Geocoding

All the shorelines generated by processing the input dataset are available in the Master SAR geometry and are all co-registered at sub-pixel level. Additionally, the results can be projected in geographic coordinates. Interestingly, the conversion from SAR coordinates to geographic coordinates is performed only for the Master geometry since all the shorelines are precisely co-registered on it. In this way, apart from reducing the computational cost of the geocoding (which is performed only for one image), we also avoid the random perturbation of geocoding errors that would result if each single image of the dataset was geocoded independently. The geolocation of the Master image is performed by taking into account the atmospheric propagation delay correction which is estimated through Numerical Weather Modeling (NWM) and is integrated into the coastal monitoring system in order to ensure a geolocation accuracy close to or better than the spatial resolution of the SAR images [67,68]. Finally, the extracted land-sea interface is further precisely geolocated using Ground Control Points (GCP).

### 2.5. Tidal Correction

After precise geocoding, land-sea boundaries extracted from SAR images have to be corrected for the effect of tidal levels and a datum-based shoreline can be defined. Neglecting tidal effects along low slope sandy beaches could lead to significant errors in evolution assessment, since high and low tide shoreline variation can be remarkable. Therefore, land-sea interface extracted from the images needs a final refinement, considering tidal oscillations, in order to be referred at 0 m above sea level. This refinement step requires a high resolution Digital Elevation Model (DEM) in the intertidal zone in order to evaluate slope along shore-perpendicular transects and tide values recorded by a tidal station in a near location. HRTI-5 is the DEM product level recommended for this correction [69].

## 3. Case Studies

**Figure 2:**
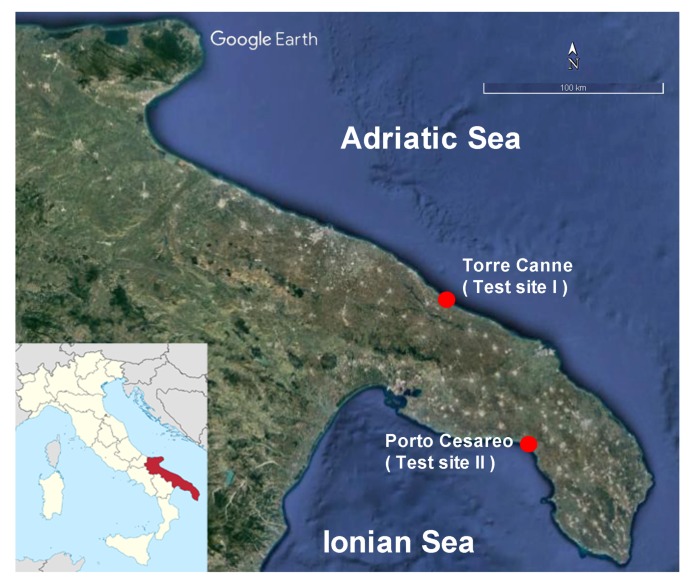
Selected test sites: Torre Canne and Porto Cesareo, both located in Southern Italy. The optical image is from GoogleEarthTM.

The proposed approach has been tested in two target areas located along the Apulian coastline (Figure 2): the first one, Torre Canne, is located along the Adriatic coast whereas the second one, Porto Cesareo, lies along the Ionian coast.

Both areas show a low-slope sandy beach affected by a remarkable erosive process that has significantly reduced the average emerged beach width. In both cases, human activities, started in the 1960s during Italian economic development, were significantly increased in recent decades and the increased land exploitation has brought a lack of sediment in the littoral cell, triggering a massive erosive trend. Due to the shoreline regression phenomena and their popularity, the selected areas have been constantly monitored for many years, so that high quality topographic and meteorological data are available in both locations.

Since 2004 several wide coastal monitoring programmes supported by the European Union (POR 2000–2006 and FESR 2007–2013 funds) have been carried out in Apulia region, allowing to obtain a detailed picture of the coastal trends and a huge database for scientific research. Those programmes included many actions: bathymetric, topographic and sedimentologic field campaigns, aerial and LIDAR surveys, real-time shoreline monitoring using webcams [14,16,28,29] and a deployment of a meteomarine network (Apulia Region Meteomarine Network, hereinafter referred to as “SIMOP”) [70,71,72]. The availability of marine weather measures turns out to be extremely helpful for accurate selection of SAR acquisitions available in the CSK archive, based on a preliminary check on the sea weather conditions at the acquisition time. All the available field data collected in the target sites are listed in Table 1.

The examined SAR images are all HH (horizontal transmitting, horizontal receiving) polarized and captured by the SAR1 satellite of the CSK constellation in Stripmap HIMAGE mode (spatial resolution: 3×3 m${}^{2}$) acquired in the framework of the MapItaly plan. The horizontal polarization is generally preferred to the vertical one since it ensures the largest contrast of the backscattering coefficient between the beach and the sea of the polarization modes [61].

A 14-km long coastal stretch has been selected as test site I, between towns of Torre Canne and Savelletri. The nearest tide gauge is located in the harbour of Bari, belonging to the Italian National Tide gauge Network (hereinafter referred to as “RMN”) and wave data are collected by a wave buoy moored 15 km north of the site which is part of the Italian Data Buoy Network (hereinafter referred to as “RON”) [73]. The H4-05 dataset of CSK acquisitions, spanning from March 2010 to May 2013, contains images acquired at around 16:52 (UTC time), along right-descending passes (Table 2).

The second experimental case study (Test site II) is located along a very popular beach (Bacino Grande) in the southern part of the Porto Cesareo municipality. It is a 3000 m long sandy beach, backed by a sub-parallel coastline dune ridge and oriented W-E. Since 2006, the area of interest has been constantly monitored by the SIMOP network, that includes: (i) an anemometric station, (ii) a tide gauge located a few hundred meters towards the South, and (iii) a wave buoy moored offshore in the Gulf of Taranto, about 60 km north of Porto Cesareo. The examined SAR images are part of two distinct CSK data takes, with an off-nadir angle of 24.13${}^{\circ }$ and 24.67${}^{\circ }$ (beams H4-01 and H4-02, respectively) and along ascending orbits (Table 2). The H4-01 dataset spans the period from 2009 to 2011, whereas H4-02 dataset was acquired between 2010 and 2015.

## 4. Results

From the CSK catalogue, two Stripmap HIMAGE acquisitions datasets, covering the target regions, have been selected according to the wind and wave conditions provided by the nearest marine observing stations from SIMOP, RON and RMN. When observed data were unavailable, that preliminary selection step has been performed exploiting ERA-Interim Re-Analysis dataset from the European Centre for Medium-Range Weather Forecasts (ECMWF) [74].

The first selected dataset (Test Site I) contains 19 images, spanning from 2010 to 2013, whereas the second one (Test Site II) contains 38 CSK images acquired between 2009 and 2015. Remotely sensed and in situ data, provided as input to the COSMO-Beach processing chain used for validating the experimental results, are listed in Table 1.

**Figure 3:**
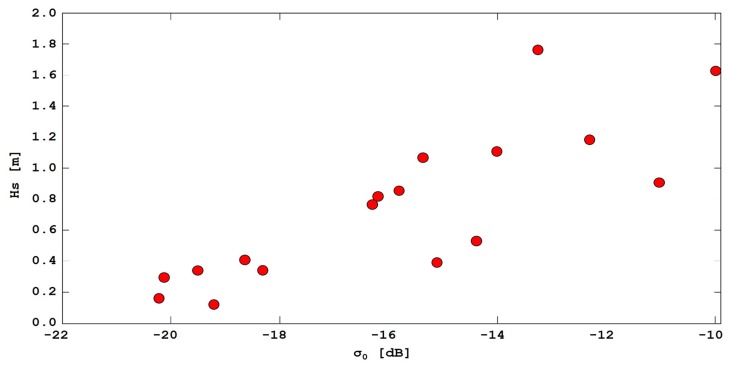
Scatter plot showing the correlation between the mean backscattering coefficient (computed offshore) and the significant wave height measured by a wave buoy (Test Site I).

Taking into account that, in case of calm sea conditions, the surface of the sea appears as a very homogeneous region characterized by rather low amplitude values, mean and standard deviation of the amplitude values have been calculated in a sub-region, located offshore the examined beach, and extracted from the filtered data, and their relationship with recorded wave heights has been investigated. The calm sea is characterized, as expected, by low backscattering coefficient values, whereas in rough sea conditions $\sigma_{0}$ values grow considerably (Figure 3).

Sea conditions can be likely distinguished using the average amplitude values: it was observed that when the backscattering coefficient exceeded a threshold value equal to −19 dB, images could certainly be discarded, since acquired in conditions of rough seas with significant wave heights ($H_{s}$), typically greater than 0.5 m. These results are in good agreement with results published in the recent literature for TSX constellation, i.e., the other X-band satellite SAR mission, which confirmed that the reflectivity of water surfaces in light wind conditions are less than −19 dB [75].

This step would allow to select part of the SAR datasets for further analysis when field data or high resolution wave model data are not available.

**Figure 4:**
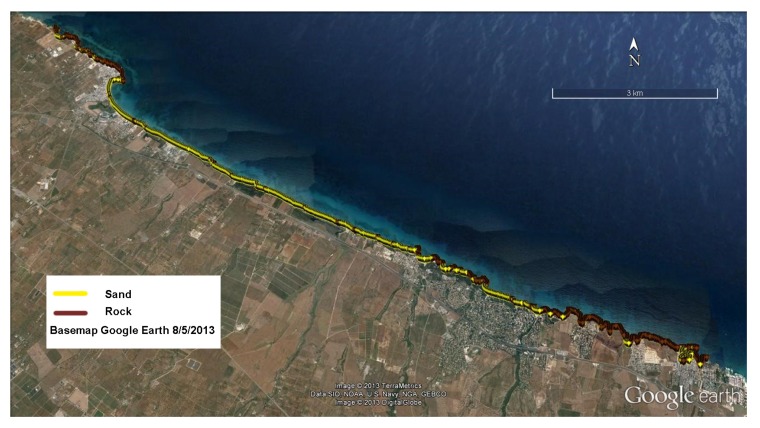
Coastal type classification over Test site I: rocky and sandy stretches are marked in brown and yellow, respectively.

After filtering out noisy images, the selected SAR images were preprocessed and precisely geocoded thanks to the availability of high quality LIDAR surveys covering both areas of interest (AOI). Concerning the use of the interferometric coherence for coastal type classification, multiple acquisition pairs have been selected, with small temporal and geometric baseline in order to minimize temporal and spatial decorrelation noise sources. An average coherence map has been hence computed for minimizing time-uncorrelated fluctuations, allowing shoreline classification (Figure 4).

Very good results have been achieved in coastal type discrimination (with a 90% success rate for Test site I and close to 100% for Test site II), as proved by aerial photos provided by the Maritime State Office of Apulia Region. The coastline classification has also been fruitfully exploited for the application of the segmentation algorithm in the next step.

**Figure 5:**
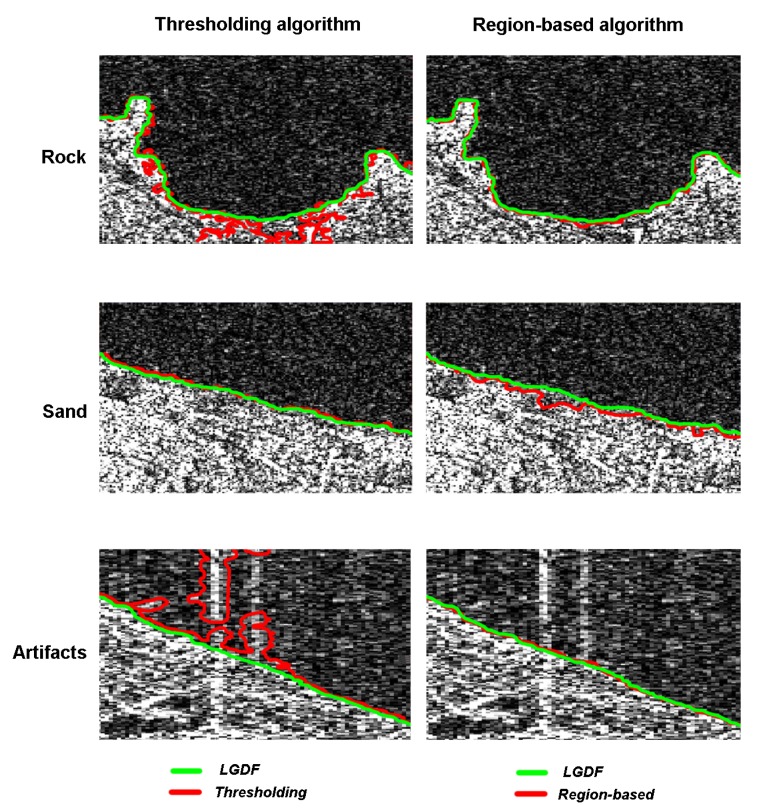
Segmentation results using thresholding, region-based algorithm and LGDF in the three examined sub-sites (rock coast, sandy coast and artifacts).

Three different segmentation algorithms have been tested on three 165×130 pixel matrices cut out of the original image, each of them representing a different coastal type (rock and sand) and one of them containing artifacts. In this step the performance of the tested segmentation algorithms has been visually inspected, overlaying the extracted shoreline on the original SAR image. In Figure 5, the land-sea interfaces extracted in the three sub-sites using three different segmentation approaches are displayed with different colors (red line for thresholding and region based, green line for LGDF). It can be seen from Figure 5 that SAR images have been correctly segmented in all the sub-sites, even on the sandy coast, by the LGDF algorithm, which is able to extract a ”visually correct” land-sea interface. The results of all tests performed have been classified into two categories (Table 3): (i) Good if the SAR land-sea interface has no anomalies and is close to the visual boundary, and (ii) Poor if the SAR land-sea interface presents anomalies or significantly departs from the visual boundary. As reported in Table 3, LGDF has been the only method capable of providing accurate coastline for both the scenarios and to be also robust to handle SAR intensity artefacts. Since it is an adaptive method capable of exploiting local variability of the SAR images, it has been proven to be reliable and efficient even in the case of SAR images with high intensity dispersion and power noise. Hence, the LGDF approach has been clearly preferred over the other two and it has been integrated in the processing chain for land-sea boundary extraction.

**Figure 6:**
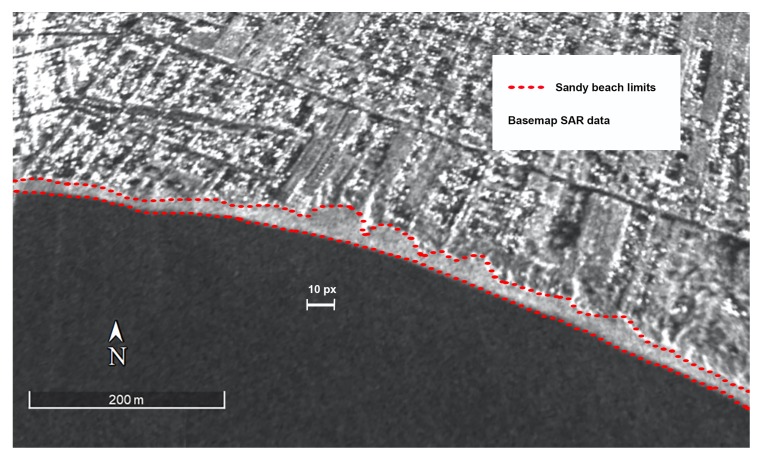
Synthetic Aperture Radar (SAR) Pixels belonging to sandy beach region.

The LGDF algorithm accuracy for land-sea interface extraction in the sandy areas have been tested on two wide sandy beaches, because in the case of narrow beaches, where beach width is smaller or equal to spatial resolution, the backscattering of the sand portion is indistinguishable from SAR echo signal of structures delimiting the beach. In selected SAR images, both the sandy beaches are represented by a good number of pixels (about 10 pixels in the widest part of the beach) even during the winter season (Figure 6). For each test site, a SAR acquisition has been taken for the segmentation assessment, selecting optimal weather and sea conditions (calm sea conditions and light winds) in the off-season in order to avoid the crowded coasts.

Each SAR image has been segmented using an LGDF approach in order to extract the instantaneous shoreline. The LGDF algorithm, initialized in the first approximation by a “coarse” line, extracted applying a global thresholding, iteratively calculates, for subsequent steps, the dry/wet separation line using different segmentation parameters according to the coastal type. Extracted shorelines were affected by tidal fluctuations; they have been hence corrected by taking into account sea level values recorded by nearest tide stations and the local slopes derived from the available high resolution DEMs, and then referred to 0 m above sea level.

During both experiments, an accuracy assessment of shorelines extracted by the COSMO-Beach system has been performed by comparing the outcomes of the processing chain to ground truths collected through GPS survey campaigns carried out simultaneously with the SAR acquisition. The surveys have been conducted using two high accuracy GPS receivers (Leica GX1230) operating in Real Time Kinematic (RTK) mode and real-time corrected through the permanent GNSS network of the Apulia Region. Within the in situ campaigns, regularly spaced shore-perpendicular transects have been surveyed and the 0 m contour has been extrapolated.

**Figure 7:**
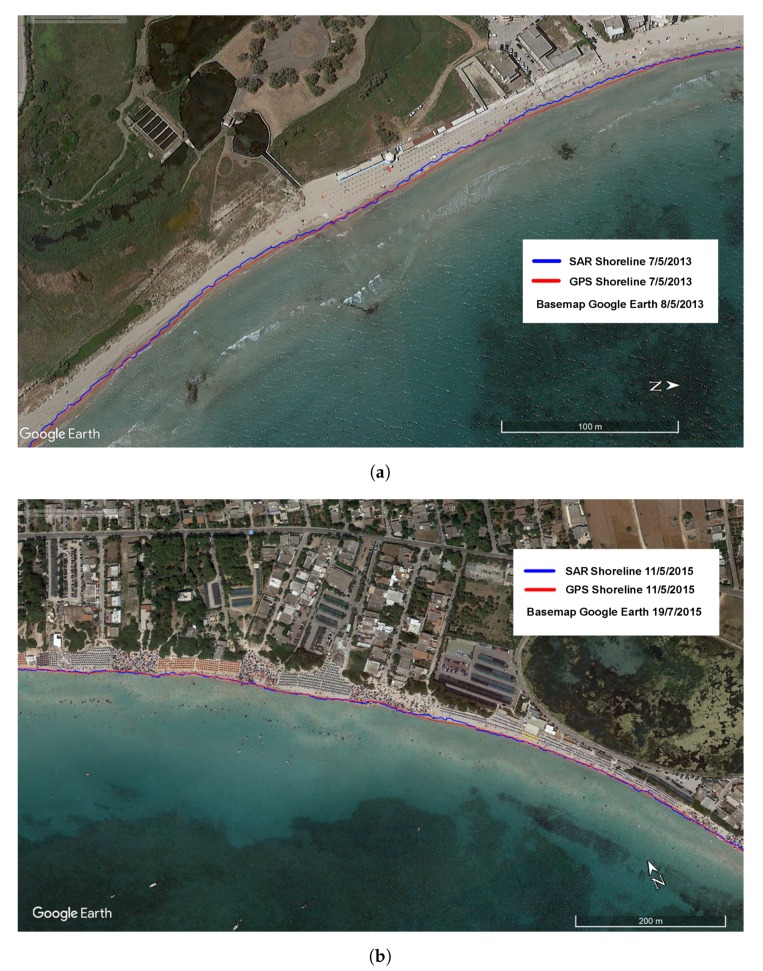
Accuracy assessment of SAR extracted shoreline: (**a**) Test site I; (**b**) Test site II.

The comparison between the corrected SAR shorelines and the observed 0 m GPS contour highlights a good performance in coastline detection using the proposed procedure (Figure 7a,b). For the statistical error analysis, 50 transects in test site I and 160 transects in test site II, located at a fixed distance of 10 m between each other, have been considered.

The overall performances of the LGDF algorithm have been estimated using a series of statistical indicators. Mean error, standard deviation and Root Mean Square Error (RMSE) have been calculated for the sample of distances between SAR and GPS shorelines for each transect. Results have shown an overall mapping accuracy (RMSE) of 1.35 m for Test Site I and 2.2 m for Test Site II, i.e., lower than image pixel resolution (Table 4).

The algorithm performance has also been assessed using a criterion based on neighbourhood pixels [40], estimating the pixel distance between the SAR shoreline and the GPS shoreline (Table 5) The accuracy analysis carried out on Test Site I shows that 87% of extracted shoreline segments lie within 1 pixel distance from the surveyed shoreline, and, with a tolerance of 2 pixels, the accuracy rises to 100%. Test Site II experiment, even with a greater number of transects, has achieved similar results: 85% of SAR coastline lies within 1 pixel distance from the GPS coastline, 98% of SAR coastline has a tolerance of 2 pixels, and the accuracy rises to 100% with a tolerance of 3 pixels.

**Figure 8:**
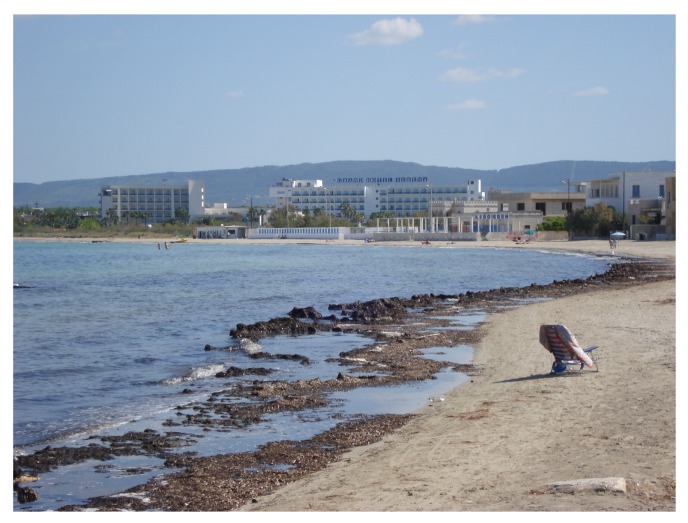
Organic deposits of *Posidonia oceanica* along the coastline during SAR acquisition.

It has to be highlighted that these results have been achieved by excluding, from the statistical analysis, transects where organic deposits of *Posidonia oceanica* were detected during on-site inspections. Larger errors have been detected only over those areas, since the intensity of the radar signal backscattered by the stockpiles hinders the reliable estimation of the shoreline through any segmentation algorithm (Figure 8). In such cases, as expected, the extracted land-sea interface lies along the algal deposit on the shore in the zone extending seaward from the “real” shoreline.

**Figure 9:**
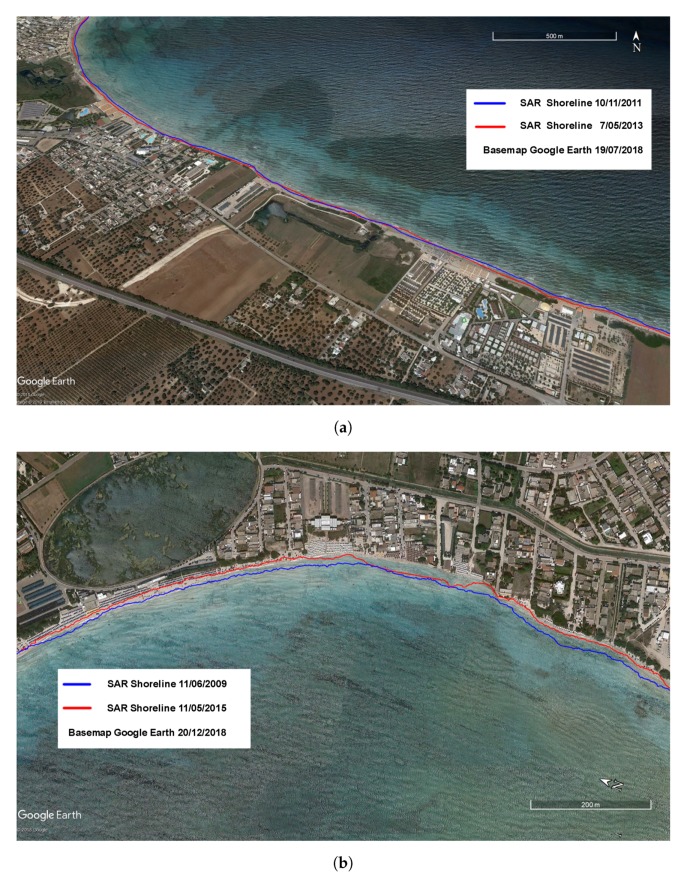
Shoreline changes detected from SAR images (blue line for the oldest SAR shoreline, red line for the most recent): (**a**) Test site I (Porto Cesareo); (**b**) Test site II (Torre Canne).

After the accuracy assessment of shoreline detection, the coastal evolution trend has been analyzed in both target areas. Shorelines have been extracted from each available SAR image and have been corrected taking into account the tidal oscillations. Shoreline movements have been measured along user-specified orthogonal transects and rates-of-change and associated statistics have been estimated. During the investigated time periods, both test sites were erosion-prone areas: the diachronic shoreline analysis revealed a maximum retreat of 24 m from 2011 to 2013 in Test Site I (Figure 9a), and a shoreline erosion up to 20 m from 2009 to 2015 in Test Site II (Figure 9b).

A comparison between SAR extracted shorelines and 2018 satellite maps (Figure 9a,b) reveals that beach erosion in both examined areas still occurs, attesting the need for a continuous coastal monitoring system for coastal risk assessment.

## 5. Discussion

A coastal monitoring system requires some key characteristics such as cost effectiveness, high-speed data acquisition and processing and the accuracy of the collected information. Traditional methods for coastal monitoring, generally ensuring higher accuracy than EO systems, determine a significant financial burden due to the extension of the areas, the survey frequency and the long-term continuity.

Since the aim of our research has been to set up an operational tool for coastal observation ensuring high accuracy, the experimental analysis has been carried out on CSK SAR data due to their high spatial resolution, low-cost, short revisit period and operational continuity. Moreover, the CSK satellite constellation has been operating since 2007, providing a large archive of SAR images that can be exploited for short and long-term analysis. The fast revisit/response time makes it possible to employ these data even for risk situation assessment. Besides, COSMO-SkyMed Second Generation is planned to become operational in 2019–2020, thus ensuring continuity of service for many more years. Currently, there are very few SAR satellite missions that combine all these features.

The direct comparison of accuracy results in shoreline extraction with previous studies is not straightforward since they depend on several factors including sensor characteristics, spatial resolution, sensor acquisition geometry, shoreline morphology, sea state, and processing methodology [76]. Moreover, it has to be underlined that in most studies the algorithm performance is estimated by an overall eye inspection [42,43,50] or a comparison with a manually traced coastline [45,48,77]. Few studies comply with ground truth data for accuracy assessment [46,47,51,78]. Focusing attention only on shoreline identification performance from CSK data estimated by comparing the extracted coastline with ground truths, our experiments are consistent with previous results [47], that found accuracies of 1 pixel for shorelines extracted by the CSK Stripmap HIMAGE data using a procedure based on image segmentation using locally adaptive thresholding method. CSK polarimetric data produce less accurate results in shoreline detection than those that can be achieved using CSK Stripmap images, providing a 7 pixel accuracy [51].

Interesting results have also been reported in the recent literature with high-resolution X-band SAR images acquired by the TSX mission. In particular, Vandebroek et al. [78] monitored a beach renourishment in the Netherlands. Thanks to the high accuracy of the timing information and orbital state vectors, TSX allows a sub-decimetric ranging accuracy and a high-quality geocoding [79,80], thus potentially ensuring a very precise georeferencing of the shoreline. Nevertheless, the authors have stated in [78] that the errors in the shoreline estimate may amount to some tens of meters, which is well above the TSX spatial resolution (∼3 m).

Although the spatial resolutions of CSK and TSX stripmap data are similar, the results detailed in [78] are not directly comparable with the outcomes of the present study, because of the very different settings of the selected test sites. In particular, the significant discrepancies between the performances achieved in [78] and in the present study, respectively, can be explained in terms of different environmental conditions, such as meteorological, tidal and topographic factors among the areas of interest.

In particular, results reported in [78] are heavily affected by the local exposure of the test site, a mega scale beach renourishment (known as “Sand Motor”) on the Dutch coast facing the North Sea, and exposed to severe winds, wave and tide conditions. Instead, tidal oscillations, wind and wave climate conditions are significantly milder in the areas selected for the present study (i.e., Torre Canne and Porto Cesareo), thus reducing the uncertainty in the still sea level. By selecting only images acquired in the most favorable weather conditions, wind and wave setup and also wave run-up have been neglected in the present study.

Moreover, even by assuming a comparable uncertainty in the measurement of the water level, the errors in the shoreline detection are expected to be significantly affected by the local slope of the shoreline: the higher the local slope, the better the accuracy of the tidal correction. The slope of the Dutch test site is much lower than the slope of the two Apulian coastal areas. Finally, SAR backscattering should be further investigated both in the intertidal zone and in areas covered by only a few centimeters of water. This further highlights the influence that environmental parameters play in the coastline extraction.

The main limitation of the presented integrated approach is related to the nature of the radar data. Unlike optical images, SAR images are not straightforward to be visually interpreted. As an example, the algorithm cannot recognize objects along the coast, as in the case of *Posidonia oceanica* blocks, because as external elements they could not be masked out in the segmentation process. Since they can affect the backscattering values in SAR images, their occurrence can hinder the accurate detection of the shoreline through SAR data segmentation analysis.

COSMO-Beach could be successfully applied to other coastal areas because it does not depend on the location of coastal areas, but it has to be highlighted that the performance in shoreline detection is strongly linked to the quality and quantity of available field and remotely sensed data, so the “one-pixel” potential accuracy can be achieved as long as all the image quality checks have been satisfied and high accuracy in situ observed data are available.

The proposed procedure for beach monitoring could be also effectively extended to SAR data provided by other European Space Agency missions released in the framework of the Copernicus Earth Observation programme. Interestingly, Sentinel-1 interferometric data can reach a revisit time of one image every six days, thus allowing a quasi real-time monitoring but their use is limited by spatial resolution (5×25 m${}^{2}$ in Interferometric Wide Swath mode). The use of these low-resolution images would likely allow the monitoring of beaches presenting relevant shoreline evolution rates (sand spits, river deltas) rather than beach environments having a small dynamic range.

## 6. Conclusions

An integrated approach of remotely sensed and in-situ data has been presented, aimed at coastal observation on both the local and regional scale for short and long-term analysis. A semi-automatic coastal monitoring system, known as COSMO-Beach, has been implemented to detect and analyze the coastline from Cosmo-SkyMed Stripmap HIMAGE data exploiting a mixed radiometric/interferometric approach. The research conducted on the COSMO-SkyMed SAR images has shown that this data satisfactorily meets coastal monitoring requirements: (i) a large archive of low-cost SAR images with a short revisit period and operational continuity; (ii) a segmentation processing can be quite easily implemented with a low computational time and (iii) a good performance in shoreline detection ensuring an accuracy lower than image pixel resolution.

The segmentation stage adopts a two-step processing procedure: a coarse shoreline, detected using a thresholding method, feeds an Active Contour Model (LGDF) that extracts a fine resolution land-sea interface. A good performance in shoreline detection has been achieved ensuring a high sub-pixel. The whole processing chain is fully automated except the fine segmentation step that requires an operator intervention in cases of anomalies in the extracted features. The integration of remote sensed and in situ data tested in this research has proven to be effective for an accurate estimation of the shoreline and for the study of its dynamics, allowing: (i) selection of high quality SAR images, (ii) precise geocoding, (iii) tidal correction, and (iv) shoreline accuracy assessment.

## Figures and Tables

**Table 1:** Remote sensed and field data selected for the experimental analysis over the two test sites.

	Test Site I	Test Site II
SAR dataset	19 CSK Stripmap HIMAGE	38 CSK Stripmap HIMAGE
DEM	LIDAR DEM 2008 1 m	LIDAR DEM 2008 1 m
	(POR PUGLIA 2000–2006)	(POR PUGLIA 2000–2006)
Aerial photos	Maritime State Office (Apulia Region)	Maritime State Office (Apulia Region)
Waves	RON Wave Buoy (Monopoli)	SIMOP Wave Buoy (Taranto)
Tides	RMN Tidal station (Bari)	SIMOP Tidal station (Porto Cesareo)
GPS survey	60 GPS transects spaced 10 m	50 GPS transects spaced 10 m

**Table 2:** COSMO-SkyMed (CSK) dataset characteristics.

	CSK_H4-05_HH_RD_009	CSK_H4-01_HH_RA_009	CSK_H4-02_HH_RA_009
Instrument Mode	STR_HIMAGE	STR_HIMAGE	STR_HIMAGE
Polarization	HH	HH	HH
Look Side	right	right	right
Pass Direction	D	A	A
Track	9	209	209
Beam ID	H4-05	H4-01	H4-02
Off Nadir Angle	30620	24130	24670
Satellite ID	SAR1	SAR1	SAR1

**Table 3:** Performances of different segmentation algorithms on the selected test site.

Algorithm	Rocky Coast	Sandy Coast	Artifact
Thresholding	GOOD	GOOD	POOR
Region-based	GOOD	POOR	GOOD
LGDF	GOOD	GOOD	GOOD

**Table 4:** Shoreline accuracy assessment.

Test Site	Mean (m)	Standard Deviation (m)	RMSE (m)
Test Site I	1.12	0.78	1.35
Test Site II	1.36	1.47	2.2

**Table 5:** Algorithm performance for different neighborhood sizes.

	Accuracy		
**Test Site**	**<1 Pixel Distance**	**1–2 Pixel Distance**	**2–3 Pixel Distance**
Test Site I	87%	100%	100%
Test Site II	85%	98%	100%
